# Pseudo-syndrome de Demons Meigs secondaire à un cystadénome séreux de l'ovaire: cas clinique

**DOI:** 10.11604/pamj.2019.33.11.18128

**Published:** 2019-05-07

**Authors:** Mouhcin Daoudi, Laila Herrak, Mustapha El Ftouh, Leila Achachi

**Affiliations:** 1 Service de Pneumologie, CHU Avicenne, Rabat, Maroc

**Keywords:** Pseudo syndrome de Demons Meigs, tumeur ovarienne, pleurésie, ascite, résolution des épanchements après chirurgie, Pseudo-Meigs syndrome, ovarian tumor, pleurisy, ascites, resolution of effusions after surgery

## Abstract

Le pseudo-syndrome de Demons Meigs, associe une tumeur ovarienne, bénigne (tous types histologiques inclus) ou maligne (ovarienne primitive ou métastase ovarienne d'un autre primitif) ou une tumeur pelvienne (non nécessairement ovarienne, utérine par exemple), à une ascite et à une pleurésie (non métastatiques en cas de tumeur maligne), ces épanchements disparaissent après résection de la tumeur. Une patiente de trente-sept ans a été admise dans notre service pour une dyspnée et un point de côté gauche. Les examens radiologiques ont objectivé une pleurésie gauche de moyenne abondance, une ascite de faible abondance et une masse pelvienne. L'exploration chirurgicale a révélé une tumeur ovarienne, après ablation, la pleurésie s'était résolue spontanément. C'est dire l'intérêt, en pneumologie, de penser à ce syndrome chez une femme qui se présente pour une pleurésie dont le bilan étiologique demeure négatif, et de prescrire un examen simple qui est l'échographie abdomino-pelvienne permettant une orientation diagnostique.

## Abstract (en)

Pseudo-Meigs syndrome combines a benign (all histological types are included) or malignant (primitive ovarian tumor or ovarian metastasis from another primitive tumor) ovarian tumor or a pelvic tumor (not necessarily ovarian or uterine, for example) with ascites and pleurisy (non-metastatic in the case of malignant tumor). These effusions disappear after tumor resection. A 37-year old female patient was admitted to our Department with dyspnoea and left intercostal pain. Radiological examinations showed left pleurisy of average abundance, ascites of low abundance and a pelvic mass. Surgical exploration showed ovarian tumor. After ablation, pleurisy solved spontaneously. Of particular interest, with regard to pneumology, is that this syndrome has occurred in a woman with pleurisy whose etiological assessment was negative and that abdominopelvic ultrasound allows diagnostic orientation.

## Introduction

Le pseudo syndrome de Demons Meigs est défini par la présence chez la femme d'une tumeur ovarienne, bénigne ou maligne, ou plus rarement d'une tumeur pelvienne non ovarienne, associée à une ascite et à une pleurésie. Le mécanisme physiopathologique sous tendant la constitution de ces épanchements n'est pas exactement connu, le bilan étiologique de ces derniers reste négatif et ils régressent habituellement après la résection de la tumeur, c'est un syndrome relativement peu fréquent en pratique courante surtout en pneumologie. L'objectif de cet article est de sensibiliser le pneumologue à cette entité, qu'il doit garder à l'esprit et savoir évoquer chez une patiente ayant une pleurésie, à fortiori si elle a une tumeur ovarienne ou des antécédents gynécologiques, bien-sûr après avoir mené une enquête étiologique exhaustive écartant les étiologies les plus fréquentes.

## Patient et observation

**Figure 1:**
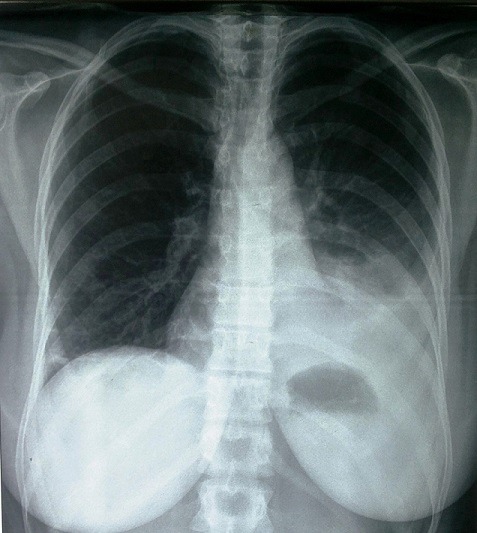
Radiographie thoracique de face montrant une pleurésie gauche de moyenne abondance

On rapporte le cas d'une patiente âgée de trente-sept ans, caucasienne, célibataire, sans aucune tare connue, et qui a comme antécédents médicaux une spanioménorrhée depuis un an. Admise dans notre service via les urgences pour un point de côté gauche, une oppression thoracique, dyspnée et fièvre, l'examen clinique a retrouvé un syndrome d'épanchement liquidien au niveau du tiers inférieur du champ pulmonaire gauche, une légère et diffuse sensibilité abdominale et une fièvre à 38°C. Une radiographie du thorax de face a été réalisée et a objectivé une pleurésie gauche de moyenne abondance (Figure 1). On a évoqué essentiellement les étiologies infectieuse et embolique vu la présentation clinique et l'âge de la patiente. Un bilan biologique standard (NFS, ionogramme, crase sanguine, sérologies virales) était normal, le dosage des d-dimères = 4900 ng/ml, un angioscanner thoracique réalisé n'a pas montré d'embolie pulmonaire proximale, son ECG était normal. Une ponction pleurale exploratrice a ramené un liquide jaune citrin, exsudatif (protido-pleurie = 51 g/l), lymphocytaire à 100%, l'examen direct ainsi que la culture étaient négatifs pour les germes usuels et pour mycobactérium tuberculosis, un GeneXpert MTB/rif et le dosage de l'adénosine désaminase dans le liquide pleural étaient négatifs. On a réalisé deux biopsies pleurales à l'aveugle (Trocart de Boutin) et on a recherché la présence de cellules malignes dans le liquide pleural à chaque fois, la biopsie a montré des remaniements inflammatoires chroniques non spécifiques et la recherche de cellules malignes était négative.

**Figure 2:**
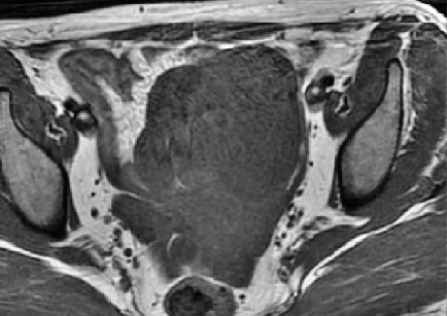
IRM pelvienne en coupe axiale montrant une masse pelvienne à paroi propre et contenant de fines cloisons en son sein: hyposignal en séquence T1

**Figure 3:**
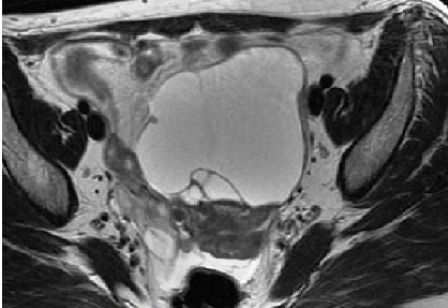
IRM pelvienne en coupe axiale montrant une masse pelvienne à paroi propre et contenant de fines cloisons en son sein: hypersignal en séquence T2

**Figure 4:**
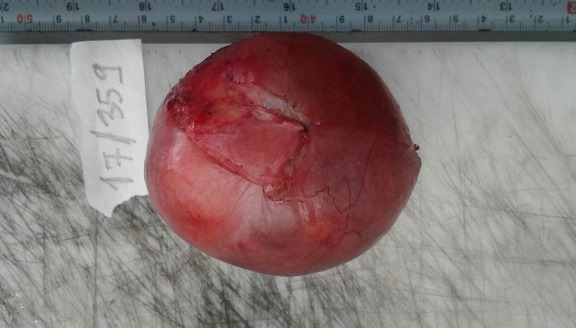
Aspect macroscopique de la pièce opératoire (tumeur ovarienne)

**Figure 5:**
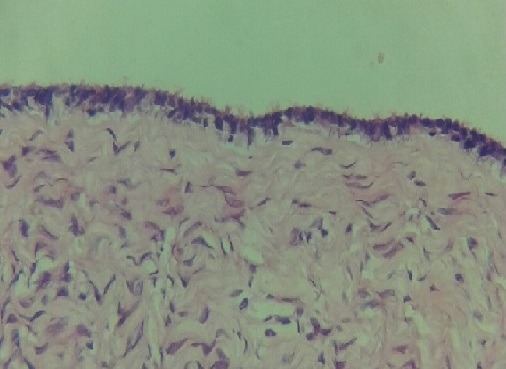
Revêtement cubique unistratifié de type séreux régulier, par place abrasé, reposant sur un tissu fibreux vascularisé ponctué de quelques éléments inflammatoires mononuclés, aspect morphologique d'un cystadénome séreux

**Figure 6:**
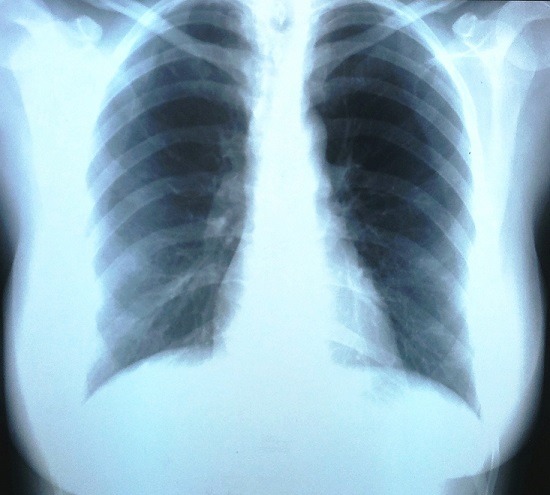
Radiographie thoracique de face normale, disparition de la pleurésie (réalisée au décours de la chirurgie)

Une fibroscopie bronchique a été également réalisée, l'aspect endoscopique était normal, des biopsies bronchiques et une étude cytobactériologique du liquide d'aspiration étaient normales. Considérant la sensibilité abdominale rapportée par la patiente et son antécédent de spanioménorrhée, la négativité du bilan réalisé jusqu'à cet instant, une échographie abdomino-pelvienne demandée a montré une collection pelvienne anéchogène, médiane, bien limitée et contenant des cloisons en son sein, on a complété par une TDM abdomino-pelvienne qui a révélé une masse pelvienne médiane, kystique mesurant 88 mm * 81 mm * 61 mm. Le radiologue a recommandé de compléter encore par une IRM abdomino-pelvienne qui a objectivé une masse pelvienne médiane mesurant 88 mm * 82 mm * 73 mm, un hydro-salpinx gauche et une ascite de faible abondance (Figure 2, Figure 3). La patiente a été transférée au service de gynécologie ou une cœlioscopie diagnostiquée a montré que cette masse prenait naissance au niveau de l'ovaire gauche, une salpingo-ovariectomie gauche a été réalisée, la pièce opératoire était une formation kystique mesurant 70 mm * 55 mm * 45 mm à paroi fine, sans végétations et d'où provenait un liquide jaune citrin (Figure 4). L'étude histo-pathologique de cette pièce opératoire a montré une formation kystique tapissée par un revêtement cubique unistratifié de type séreux régulier, par place abrasé, reposant sur un tissu fibreux vascularisé ponctué de quelques éléments inflammatoires mononuclées, le parenchyme ovarien résiduel était sans particularités, l'aspect morphologique était celui d'un cystadénome séreux (Figure 5). Une radiographie du thorax réalisée quelques jours après la chirurgie, était normale, la pleurésie s'était résolue (Figure 6).

## Discussion

Le syndrome de Demons Meigs est l'association d'une tumeur ovarienne bénigne, essentiellement un fibrome ou un thécome, d'une ascite et d'une pleurésie, ces derniers disparaissent après résection de la tumeur. Le diagnostic du pseudo syndrome de Demons Meigs repose sur les mêmes critères, la différence est que la tumeur peut être bénigne ou maligne, sans restriction concernant le type histologique de celle-ci, c'est en quelque sorte, une extension de la définition du syndrome de Demons Meigs, en cas de tumeur maligne, il faut éliminer une éventuelle atteinte péritonéale ou pleurale métastatique source d'ascite et de pleurésie avant de considérer ce diagnostic. C'est un syndrome généralement rencontré chez la femme âgée entre 40 et 60 ans, les symptômes ressentis sont dus aux épanchements présents, les examens radiologiques sont la clé du diagnostic en montrant l'existence d'une tumeur ovarienne, le traitement est chirurgical et conduit à la régression des épanchements une fois la tumeur est réséquée, la récidive est rare mais quelques cas ont été décrits [1]. En 1937, Dr Meigs a rapporté pour la première fois, sept cas de patientes ayant un fibrome ovarien avec pleurésie et ascite [2]. En 1954, Beecham a rapporté quatre cas de patientes ayant un cystadénome pseudo-mucineux de l'ovaire associé à une pleurésie et à une ascite [3]. La revue de la littérature retrouve plusieurs cas rapportés de ce syndrome associé à des tumeurs ovariennes bénignes: cystadénome pseudo-mucineux [4], cystadénome mucineux [5], hémangiome capillaire ovarien [6] ou à des tumeurs ovariennes malignes [7], un cas associé à un fibrome utérin a été décrit [8], un cas du à une métastase ovarienne d'un cancer colique a été décrit chez une patiente [9]. Ohsawa *et al*. ont rapporté un cas du à des métastases ovariennes bilatérales d'un adénocarcinome sigmoidien avec ascite et pleurésie bilatérale, l'ovariectomie bilatérale a permis la régression des épanchements même si la patiente est décédée après suite à des métastases hépatiques et osseuses de son adénocarcinome [10]. Loizzi *et al*. ont rapporté un cas de ce syndrome associé à un goitre ovarien chez une femme, le goitre ovarien faisant partie des tératomes ovariens, il s'agit d'une tumeur bénigne [11]. Cramer *et al*. ont rapporté un cas de ce syndrome associé à un carcinome papillaire séreux ovarien, avec augmentation de sécrétion d'amylase au niveau de la tumeur et des taux élevés d'amylase au niveau du liquide pleural [12]. Plusieurs théories ont été proposées pour expliquer la physiopathologie des épanchements observés dans ce syndrome, l'ascite serait due à des mécanismes comme une irritation directe du péritoine, une obstruction lymphatique, dues au développement de la tumeur, à une torsion de celle-ci ou encore à un écoulement liquidien en provenant directement, certains auteurs relient l'apparition de la pleurésie au passage du liquide d'ascite à travers les déhiscences existant au niveau du diaphragme surtout à droite, la nature biochimique de ces épanchements peut être soit un transsudat ou un exsudat [2-13].

Le cas qu'on rapporte, concerne une patiente qui a été hospitalisée dans un service de pneumologie pour une pleurésie, les étiologies les plus fréquentes dans notre contexte sont la tuberculose, les pleurésies purulentes à germes banals et les pleurésies métastatiques, l'urgence est représentée par l'embolie pulmonaire ou la pleurésie n'est qu'un symptôme. Une fois un angioscanner fait et une embolie pulmonaire éliminée (probabilité clinique intermédiaire et taux de d-dimères positif), on a réalisé une ponction pleurale exploratrice, l'aspect macroscopique était jaune citrin et l'étude biochimique a retrouvé un exsudat, on a ensuite complété par une biopsie pleurale et aspiration du liquide pour recherche de cellules malignes (50 ml de liquide pleural), tout le bilan est revenu négatif. Pour la deuxième fois, on a réalisé une biopsie pleurale et une recherche de cellules malignes dans le liquide pleural (50 ml), avec cette fois ci, le dosage de l'adénosine désaminase et la réalisation d'un GeneXpert MTB/rif dans le liquide, tout le bilan était négatif. A la lumière du bilan réalisé, on a pu écarter avec assurance une tuberculose pleurale, une pleurésie purulente, les coupes parenchymateuses de l'angioscanner n'ont pas montré de lésions pulmonaires ou pleurales, l'état général de la patiente était conservé, une origine maligne (primitive ou métastatique) était peu probable quoique non formellement éliminée. La prochaine démarche diagnostique consistait en l'indication d'une thoracoscopie médicale, cependant, la sensibilité abdominale retrouvée chez cette patiente nous a incité à réaliser une échographie pelvienne qui a objectivé l'existence d'une masse pelvienne, à ce moment-là, on a redouté la présence d'une tumeur maligne et on a approfondi l'exploration radiologique (Tomodensitométrie puis imagerie par résonance magnétique) sans pour autant en avoir la certitude, il s'agissait toujours d'une masse pelvienne avec en plus de ce qu'a montré l'échographie, d'une ascite de faible abondance associée, l'origine de cette masse pelvienne n'a pu être déterminée avec exactitude, après concertation avec les gynécologues, il a été décidé de réaliser une c'lioscopie diagnostique, la nature ovarienne de cette masse a été confirmée et une salpingo-ovariectomie gauche réalisée. Ce n'est qu'après la réception du résultat de l'étude histo-pathologique affirmant que la masse pelvienne n'était autre qu'un cystadénome ovarien, que le diagnostic de pleurésie d'origine maligne est devenu encore moins probable et que celui de pseudo syndrome de Demons Meigs a été évoqué. La réalisation d'une radiographie du thorax une semaine après la chirurgie a montré la disparition de la pleurésie, on n'a pas pu s'assurer de la régression de l'ascite qui était déjà de faible abondance (détectée uniquement à l'imagerie par résonance magnétique) vu la période post opératoire et l'impossibilité de refaire cet examen dans cette indication. Considérant les données relatées ci-dessus, on a retenu le diagnostic de pseudo syndrome de Demons Meigs chez cette patiente. Il est important, devant une pleurésie chez une femme, surtout si elle a une histoire médicale avec antécédents ou symptômes gynécologiques, d'avoir cette hypothèse diagnostique à l'esprit, particulièrement pour un pneumologue, une fois l'enquête diagnostique a éliminé les étiologies habituelles des pleurésies, il faut penser à explorer dans ce sens et prescrire les examens radiologiques utiles en commençant par un, tout simple, l'échographie.

## Conclusion

La particularité de ce travail réside dans le fait que ce syndrome est rarement rencontré, du moins en pratique clinique, dans un service de pneumologie, la quasi-totalité des cas sont rapportés par des gynécologues, on estime qu'il est inhabituel d'aboutir à ce diagnostic chez une jeune femme hospitalisée dans un service de pneumologie pour une pleurésie isolée, sans signes gynécologiques évidents, étant donné les autres étiologies de pleurésies nettement plus fréquentes dans notre contexte. Le type histologique, cystadénome séreux, parait aussi rarement associé à ce syndrome, on n'a pas retrouvé une telle association décrite dans la littérature.

## Conflits d’intérêts

Les auteurs ne déclarent aucun conflit d'intérêts.
